# Draft genome sequence of XDR *Aeromonas caviae* strain NGCRVN-08 isolated from diarrheal patient in Bangladesh

**DOI:** 10.1128/mra.01261-24

**Published:** 2025-05-08

**Authors:** Md. Mashiur Rahaman, Abdus Sadique, Aura Rahman, Fahad Khan, Jahidul Alam, Raiyan Rafsan, Salahim Abir, Nisat Tabassum, Maria Mehjabin Oishi, Aparajita Raisa Chowdhury, Ayesha Sharmin, Md. Mainul Hossain, Munirul Alam, Maqsud Hossain

**Affiliations:** 1 NSU Genome Research Institute (NGRI), North South University 54495, Dhaka, Bangladesh; 2 Department of Biochemistry & Microbiology, School of Health and Life Sciences, North South University 54495, Dhaka, Bangladesh; 3 Department of Electrical and Computer Engineering, School of Engineering & Physical Sciences (SEPS), North South University 54495, Dhaka, Bangladesh; 4 Department of Chemistry, Bangladesh University of Engineering and Technology 61750, Dhaka, Bangladesh; 5 icddr,b, International Centre for Diarrhoeal Disease Research, Dhaka, Bangladesh; Portland State University, Portland, Oregon, USA

**Keywords:** *Aeromonas caviae*, Antimicrobial resistance, Whole genome sequencing (WGS)

## Abstract

Here, we report the draft genome sequence of *Aeromonas caviae* strain NGCRVN-08, isolated from a stool sample of a diarrheal patient in Bangladesh. Most notably, this strain harbors an extensive array of antibiotic resistance genes spanning eight major drug classes, including extended-spectrum β-lactamases and multiple aminoglycoside resistance determinants.

## ANNOUNCEMENT

*Aeromonas caviae* is an emerging pathogen of significant clinical concern, associated with a spectrum of human infections including gastroenteritis, wound infections, septicemia, and respiratory tract infections (1–3). The organism’s ability to acquire and harbor multiple antibiotic resistance determinants, coupled with its ubiquitous presence in aquatic environments, makes it a potential reservoir for the spread of antimicrobial resistance genes (4, 5). The rise of multidrug-resistant *Aeromonas caviae* strains presents a critical treatment challenge, particularly in high-burden regions like Bangladesh (6).

Strain NGCRVN-08 was isolated from a diarrheal patient’s stool sample in Dhaka on 1 November 2022. One milliliter of stool was inoculated into 9 mL APW media for enrichment and incubated at 37°C for 18–20 hours and then cultured on TCBS media and incubated overnight at 37°C. A single visibly contamination-free colony was then subcultured on GA medium under the same conditions for further use. Genomic DNA was extracted from a single colony allowed to grow overnight in LB broth media at 37°C using QIAamp DNA Mini Kit (QIAGEN, Germany) following manufacturer’s protocol. For sequencing, library preparation was performed using the Nextera XT DNA Library Preparation Kit (Illumina, Inc., USA) and sequenced on an Illumina MiSeq platform using MiSeq Reagent Kit V3 (Illumina, Inc., USA) with paired-end sequencing (2 × 150) approach generating 653,215 paired-end reads totaling 156.6 Mbp. Quality control using FastQC v0.11.9 (7) and trimming with Trimmomatic v0.39 (8) (AVGQUAL 30) yielded 407,548 high-quality reads, maintaining a consistent GC content of 58%–59%. The genome was assembled using Unicycler v0.5.1 (9) with default parameters which comprises 196 contigs with a total length of 4.91 Mb, N50 of 42,735 bp, and GC content of 60.24%. The largest contig spans 298,557 bp. BUSCO analysis (Galaxy Version 5.8.0; Lineage: Gammaproteobacteria) (10, 11) revealed the genome assembly to be 98.6% complete, with 98.1% of BUSCOs present as single-copy orthologs, 0.5% duplicated, 1.4% fragmented, and 0.0% missing, indicating a high-quality genome assembly. Genome annotation using Prokka (12) identified 4,554 coding sequences, 5 rRNA genes, and 98 tRNA genes. Annotation by NCBI Prokaryotic Genome Annotation Pipeline (13) v6.8 predicted the genome to contain 4,762, out of which 4,578 are protein coding, 6 rRNA genes, and 91 tRNA genes.

Analysis using ResFinder v4.6.0 (14) revealed a potentially extensive multidrug resistance profile encompassing critical antibiotic classes. The strain carries multiple β-lactam resistance genes, including blaCTX-M-3, blaTEM-1B, and blaMOX-6, conferring resistance to multiple generations of cephalosporins, penicillins, and monobactams. Aminoglycoside resistance is mediated by aac(6′)-Ib3 and aadA2, providing protection against clinically important drugs like amikacin and tobramycin. The presence of qnrS2 confers resistance to quinolones, while TOprJ2 affects all major tetracyclines, including the last-resort antibiotic tigecycline.

Notably, the presence of the IncQ2 plasmid, confirmed by staramr (Galaxy Version 0.11.0) (11, 15), suggests potential for horizontal gene transfer. This extensive resistance profile highlights the potential of *A. caviae* to serve as a reservoir for AMR genes and underscores the importance of surveillance and appropriate antimicrobial stewardship in clinical settings. All tools used in this study were run with default parameters unless otherwise specified.

## Data Availability

The genome sequence has been deposited at DDBJ/ENA/GenBank under the accession number JBJGVZ000000000. Raw reads are available in the NCBI SRA database under the accession number SRR31323044. The BioProject and BioSample accession numbers are PRJNA1185448 and SAMN44705475, respectively.
